# Behavioral and molecular studies of quantitative differences in hygienic behavior in honeybees

**DOI:** 10.1186/s13104-016-2269-y

**Published:** 2016-10-21

**Authors:** Tanja Gempe, Silke Stach, Kaspar Bienefeld, Marianne Otte, Martin Beye

**Affiliations:** 1 Institute of Evolutionary Genetics, Heinrich Heine University Duesseldorf, Universitaetsstrasse 1, 40225 Duesseldorf, Germany; 2 Institute for Bee Research Hohen Neuendorf, Friedrich-Engels-Strasse 32, 16540 Hohen Neuendorf, Germany; 3 Fischerweg 5, 2503 Biel, Switzerland

**Keywords:** Varroa, Hygienic behavior (HB), Varroa sensitive hygiene (VSH), Gene expression, Quantitative genetic trait

## Abstract

**Background:**

Hygienic behavior (HB) enables honeybees to tolerate parasites, including infection with the parasitic mite *Varroa destructor*, and it is a well-known example of a quantitative genetic trait. The understanding of the molecular processes underpinning the quantitative differences in this behavior remains limited.

**Results:**

We performed gene expression studies in worker bees that displayed quantitative genetic differences in HB. We established a high and low genetic source of HB performance and studied the engagements into HB of single worker bees under the same environmental conditions. We found that the percentage of worker bees that engaged in a hygienic behavioral task tripled in the high versus low HB sources, thus suggesting that genetic differences may mediate differences in stimulated states to perform HB. We found 501 differently expressed genes (DEGs) in the brains of hygienic and non-hygienic performing workers in the high HB source bees, and 342 DEGs in the brains of hygienic performing worker bees, relative to the gene expression in non-hygienic worker bees from the low HB source group. “Cell surface receptor ligand signal transduction” in the high and “negative regulation of cell communication” in the low HB source were overrepresented molecular processes, suggesting that these molecular processes in the brain may play a role in the regulation of quantitative differences in HB. Moreover, only 21 HB-associated DEGs were common between the high and low HB sources.

**Conclusions:**

The better HB colony performance is primarily achieved by a high number of bees engaging in the hygienic tasks that associate with distinct molecular processes in the brain. We propose that different gene products and pathways may mediate the quantitative genetic differences of HB.

**Electronic supplementary material:**

The online version of this article (doi:10.1186/s13104-016-2269-y) contains supplementary material, which is available to authorized users.

## Background

Worker honey bees can detect and remove parasitized brood from their nest, thus reducing the damaging effects of various parasites in the colony. This so-called hygienic behavior (HB), performed by single bees in a colony, plays a major role in the overall resistance of the colony to a number of important pathogens, including *Ascosphaera apis* (which induces chalkbrood disease) [1, 2], *Paenibacillus larvae* (which causes American Foulbrood) [3, 4] and the mite *Varroa destructor* [2, 5, 6]. HB has a genetic component, as inferred by genetic crossing experiments and scoring of hygienic performance [7]. The hygienic task usually involves uncapping behavior that results in the uncapping of the diseased brood cell and removing behavior that involves the removal of the pupae from the cell [7]. *Varroa* sensitive hygiene (VSH) behaviors specifically refer to the removal of *Varroa* mite-parasitized pupae [2, 8, 9]. Quantitative genetic studies have identified genes associated with the HB and *Varroa* sensitive hygiene (VSH) behavior [10–17]. Differently expressed genes (DEGs) have been identified between high and low HB and VSH-selected honeybee lines [10, 11] and between worker bees from a high performing line either displaying VSH behavior or not display such behavior [12]. Other approaches have correlated expression levels with the quantitative performance of HB and VSH in local honeybee breeding populations [13, 14]. The assembly of genetically high and low HB performing worker bees in different proportions in a single group has enabled the identification of genes indirectly regulated by high performing social neighbors (indirect genetic effects) [15]. Backcrosses from high and low HB and VSH-selected lines have established large genetic mapping populations in which high and low HB or VSH alleles segregate. Genetic mapping of the behavioral performance of worker bees in such backcrosses has allowed for the identification of six and two quantitative trait loci (QTLs) in HB and VSH populations, respectively [16, 17]. The genomic regions and candidate genes have been identified with the help of the genomic sequence [18]. Together, these studies have generated a list of genes that have provided further information about the molecular processes associated with HB and VSH behavior, which can provide a source to detect biomarkers for marker-assisted selective breeding of disease- and parasite-tolerant bees [4, 16, 19, 20]. How the differences in the quantitative genetic traits affect gene expression and thus, the molecular state and the selection of possible markers are unknown.

In the present study, we identified HB-associated DEGs in single worker bees that originated from genetic high and low HB sources. This was achieved through crosses of bees from three colonies with a low quantitative genetic trait for HB and three colonies with a high HB trait. We scored the behavior of the workers in a common social environment and examined HB-associated DEGs from the high and the low HB source.

## Results

### Three times more worker bees from the high source group, compared with the low source group, engaged in HB

**Fig. 1:**
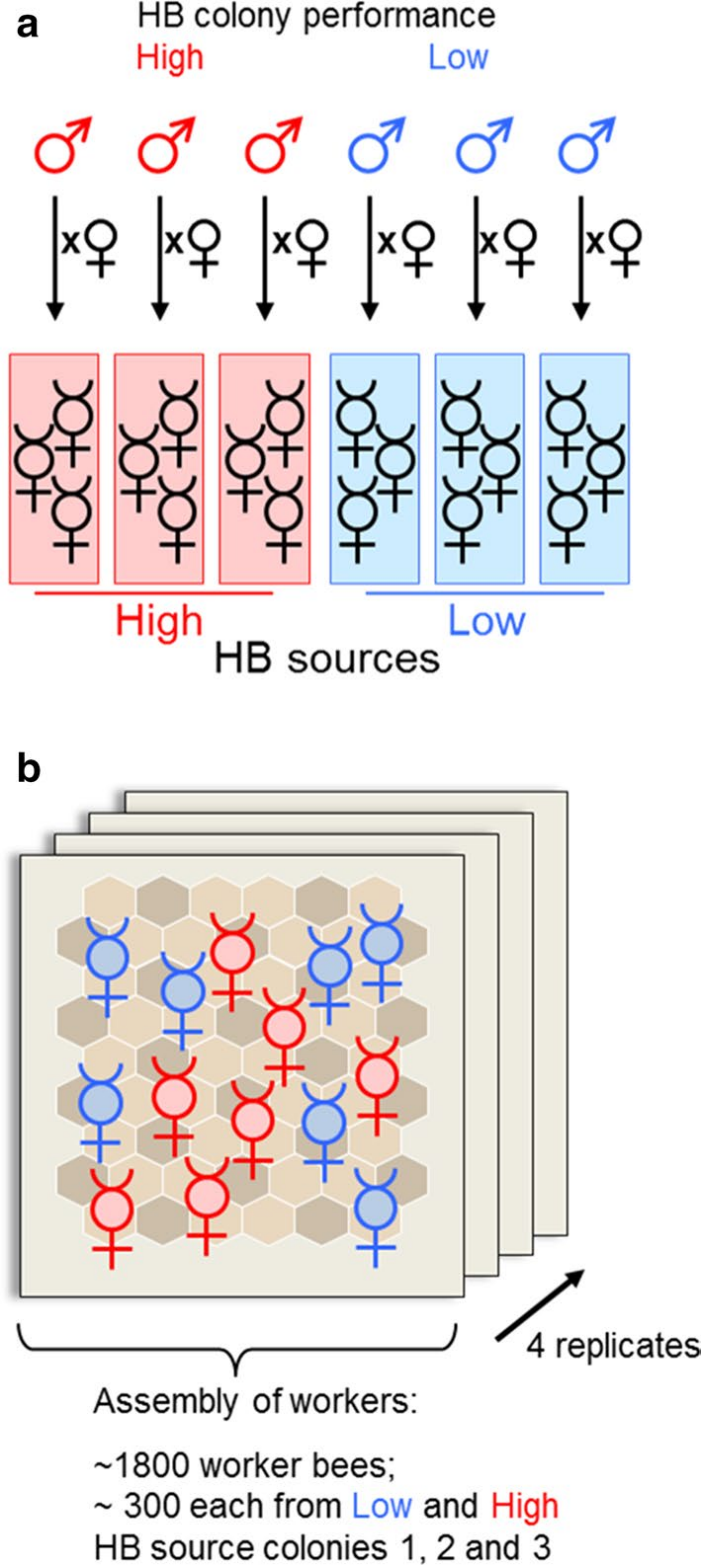
The generation of worker bees from high and low HB (hygienic behavior) sources and their assembly into a single group for the behavioral assay. **a** We generated three high HB sources and three low HB sources by crossing queens with drones that derived from colonies that had high and low breeding values for HB. The HB breeding values are derived from estimates that take into account the amount of removed dead brood in an assay (pin-test [7]). **b** Three hundred worker bees from each of the three high and three low HB sources were individually marked and assembled into a single group to score the workers’ hygienic behavioral performance with respect to pin-treated brood cells containing dead pupae. This experiment was repeated four times with different set of worker bees

**Fig. 2:**
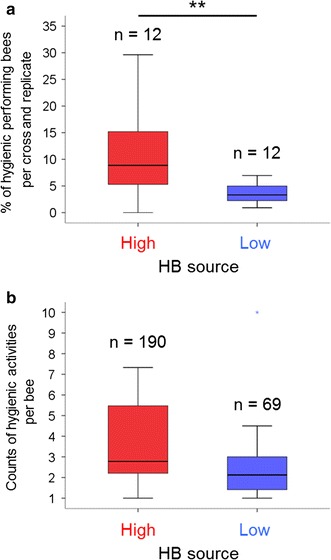
The HB activities of single worker bees derived from the high and low HB source and crosses. **a** The portion of worker bees engaging in hygienic behavioral tasks. **b** The hygienic activities per worker from the different sources and crosses. Medians were compared using the Mann–Whitney-U test, and **denotes *P* < 0.01; *P* in figure **b** was *P* = 0.19

We generated three high and three low HB performing colonies (Fig. 1) through crossings of progeny selected from an ongoing breeding program for *Varroa*-tolerant bees, as identified on the basis of overall colony performance in the removal of dead brood (pin-test [21]). To identify the workers that performed HB, we assembled 300 newly emerged worker bees from each of the three low and high HB colonies and marked each bee. At the age of 12 days old (an age at which worker bees usually can perform HB) ~1800 worker bees were confined on a brood comb containing ~33 pin-killed pupae [22]. We recorded the behavior of the worker bees by video for 12 h. We studied four biological replicate groups, resulting in a total of ~7200 worker bees included in our analysis. We obtained hygienic behavioral information (whether the worker bees engaged in uncapping or removing behavior) from 3938 worker bees. For the high HB source, we found that 8.8 % (median) of worker bees per cross and replicate (Fig. 2) engaged in HB (190 out of the 1912 worker bees). For the low HB source, 3.3 % (median) of worker bees performed HB activity (69 out the 2026 worker bees), which was significantly lower than the values observed for the worker bees from the high HB sources (*MWU*-test, *P* < 0.01, Fig. 2a). This result indicated that the portion of workers that engaged in HB among the high HB performing worker bees was nearly three times that of the worker bees from the low HB source. Because worker bees from the high and low HB colonies experienced the same environment (we assembled them into a single group), we concluded that worker bees from the high HB source displayed a higher frequency of HB engagement. However, the number of hygienic activities per worker bee was not measurably different between the high and the low HB sources (Fig. 2b). This result suggests that the average frequency in engagement of single bees in HB was not affected by the source (high vs low HB source).

### HB performing worker bees from the high and low HB sources have distinct molecular states in their brains

**Fig. 3:**
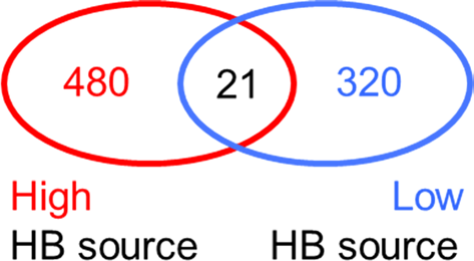
Venn diagram showing the hygienic behavior (HB)-associated genes in the brain of workers derived from the high and low HB sources

To identify molecular processes in the brain that are associated with HB in bees from the high and low HB sources, we repeatedly measured transcription profiles of 13,440 genes. We used 82 two-color microarrays that were hybridized in a loop design [23] (Additional file 1: Table S1) to identify DEGs between the different behavior and genetic source conditions. Worker bees from the three high and the three low sources were similarly represented (Additional file 1: Table S1). We identified 501 genes (3.7 % of the genes present on the microarray) that were differently expressed between the HB-performing versus the non-performing bees from the high HB source (Table 1; Additional file 2: Table S2). We identified 342 differently expressed genes (2.5 %) between the behavioral states in worker bees from the low HB source (Table 1; Additional file 3: Table S3). We observed that more genes were upregulated in bees from the high HB source than in bees from the low HB source (Table 1; χ^2^, df = 1, *P* < 0.0001), suggesting that HB-associated genes were more often upregulated in worker bees from the high HB source. We also found that 480 HB-associated genes were observed in only the high HB source, and 320 were observed in only the low HB source, whereas only 21 genes were common between the two (Fig. 3). The gene expression patterns and molecular states associated with HB markedly differ between the high and the low HB sources. Furthermore, we detected only 5 genes (Additional file 4: Table S4) that were differently expressed due to the different genetic backgrounds between the high and low HB sources, suggesting that the different genetic sources only marginally affect gene expression.

**Table 1:** Number of DEGs associated with hygienic behavior (HB) in the high and low HB sources

		# of DEGs in the HB sources^a^	
		High	Low
Upregulated	↑	339	155
Downregulated	↓	162	187
Sum	Σ	501	342

^a^ *P* < 0.01 was adjusted for multiple testing

We then performed gene ontology (GO) analysis of the 501 and 342 HB-associated genes from the two sources. Genes were assigned to their orthologs in *Drosophila melanogaster*, and functional clustering was performed using DAVID [24, 25]. For the high HB source, we found a single cluster with an enrichment score above 2, which included 11–47 genes. The GO terms “cell surface receptor ligand signal transduction” and “sensory perception of smell” were overrepresented. Proteins that were predicted to integrate into the membrane or possess an “olfactory receptor” domain or that carry glycosylation were also overrepresented (Additional file 5: Table S5). For the HB-associated genes in the low source, we found a small cluster of genes (a cluster comprising 8 to 3 genes above an enrichment score greater than 2). The GO term “negative regulation of cell communication” was overrepresented, and some of the genes within this cluster are predicted to regulate protein kinase pathways (Additional file 6: Table S6). Together, the results of the GO analysis suggest that different molecular processes in the high and the low source groups are associated with HB performance.

## Discussion

We produced three high and three low HB worker bee sources by genetic crossing. The colonies that produced the parents were selected according to the display of high or low quantitative genetic differences associated with the number of dead pupae removed from the colony in pin-tests [7]. Our results suggested that the quantitative differences in hygienic performance at the colony level were achieved by different percentages of workers engaging in the hygienic task. The percentage of worker bees engaging in a hygienic behavioral task tripled in the high versus the low HB source groups (Fig. 3), whereas the average frequency of HB engagement of a single worker was not detectably different between the two sources. These results suggested that better HB colony performance is primarily achieved by a high number of bees engaging in the hygienic tasks, rather than by a subset of highly specialized worker bees repeatedly performing that task. This finding is consistent with the view that all bees in a colony are capable of performing HB [26]. However, in the high HB backcross, more worker bees were stimulated to perform HB, possibly because of the lower level of stimulus perception and processing (see below) through olfaction [27–29]. Hence, the partitioning among hygienic and non-hygienic tasks can be modulated by various genetic propensities for performing HB, consistent with the response threshold model of task allocation [30, 31]. The proposed function of the DEGs associated with HB performance further suggests that further downstream neuronal processes in addition to perception using the antennae may also affect HB. We found that in the brain, signal transduction in the high HB source, and cell communication was overrepresented in the low HB source were overrepresented molecular processes. We speculate that these molecular processes of the brain may influence the quantitative genetic differences of HB at the phenotypic level.

Further, we found that the molecular processes in the brain associated with quantitative genetic differences involved in the engagement in HB can be markedly different. From the 501 and 342 HB-associated DEGs that we identified in the two sources, only 21 genes were common between the two groups of bees. Furthermore, GO analysis revealed no overlap in the assigned GO terms and gene functions for the two sets of DEGs. We conclude that the molecular states in the brain that associate with HB worker performance are variable. We speculate that some of the molecular patterns that are confined to the HB worker source may relate to the more stimulated state of those worker bees coming from the HB sources. However, it remains unclear whether those genes and their gene products are the cause of the higher stimulated state or are the product of the hygienic behavioral engagement. Irrespective of the underlying cause, our results suggest that the relationship between gene products and the quantitative differences in worker engagement into the hygienic task may be associated with distinct molecular processes.

We next investigated whether the DEGs that we identified have also been identified in other HB and VSH studies (Table 2; Additional file 7: Table S7). We speculated that more of the DEGs from the high HB source group in our study would match the gene candidates in previous studies than those DEGs from the low HB source because they should associate with the high HB or VSH performance evaluated in previous studies. We found that 7 % (34 genes) of our high and 9 % (29) of our low source DEGs have been previously associated with HB (Table 2). Hence, the DEGs in the high HB source (χ^2^, df = 1, *P* > 0.39) were not overrepresented among the genes reported in previous studies. A possible explanation for this discrepancy is that the design of the experiments, the characterized genetic differences (QTL versus gene expression differences (DEGs)) and the scored phenotypes differed among studies. Some DEGs studies have compared the DEGs in pupae or in adult brains between selected VSH high and low lines [10, 11]. One study has identified DEGs in a selected VSH line by comparing DEGs in the antennae, which were collected from workers that were or were not performing VSH behaviors [12]. Others have correlated expression levels with the quantitative HB and VSH performance in a local breeding population [13, 14]. One study has identified DEGs associated with relatively high HB activity that were induced by the social neighbors (indirect genetic effects) [15]. QTL mapping identified different genomic loci that associated with HB or VSH [16, 17]. In this study we found that the DEGs and the GO terms differed between our low and high performing sources. Only few genes were shared despite the fact that the same methods were used. The different molecular states in worker brains (large set of different DEGs and associated GO terms in high and low HB performing worker bees) suggest that possibly different molecular routes are involved in the regulation of quantitative differences in HB behavior. If validated by further experiments, a different molecular basis for quantitative differences in HB would have broader implications for the evolutionary origin of behavioral variants and the molecular regulation of social behaviors. Knowledge of the different genetic and molecular underpinnings would also strongly affect the selective breeding strategy and the identification of biomarkers [4, 16, 20].

**Table 2:** The HB associated DEGs found also in previous HB and VSH studies

This study		Previous studies						
HB sources	Sum of common genes	Gempe 2012 brain M^a^	Navajas 2008 pupae M^a^	LeConte 2011 brain M^a^	Tsuruda 2012 Q^b^	Oxley 2010 Q^b^	Parker 2012 (antenna, larvae P^c^	Mondet 2015 antenna R^d^
High	34 (7 %)	20 (3 %)	2 (2 %)	3 (16 %)	2 (2 %)	1 (3 %)	0	9 (5 %)
Low	29 (9 %)	20 (3 %)	2 (2 %)	1 (5 %)	2 (2 %)	2 (7 %)	0	4 (2 %)

^a^Microarray analysis

^b^QTL analysis

^c^Proteome analysis

^d^RNAseq analysis

## Conclusions

The better HB colony performance is primarily achieved by a high number of bees engaging in the hygienic tasks, rather than by a subset of highly specialized worker bees repeatedly performing that task. We also provided evidence that molecular processes in the brain (in addition to perception using the antennae) may affect quantitative differences of HB. We found distinct molecular states in the brains that were associated with quantitative differences in HB. Signal transduction was an overrepresented molecular process in the brain of the high HB source, and cell communication was overrepresented molecular process in those of the low HB source. Different molecular underpinnings for quantitative differences in HB may have broad implications for selective breeding strategies and for understanding of the evolution and molecular control of social behaviors in honeybees.

## Methods

### Sources of honeybees

We identified colonies (*Apis mellifera*) that displayed varying degrees of HB performance, using a large database from an ongoing breeding program (www.beebreed.eu). The breeding program calculate breeding values for HB based on group performance using the pin-test [32]. The breeding values were estimated by using the BLUP (Best Linear Unbiased Method) approach [33]. We crossed three queens with a single drone that derived from colonies that had the highest breeding values for HB in our sample (values see Additional file 7: Table S7). The offspring of those three crosses established the workers in the high HB source group in our experiment. We also crossed three queens with drones that derived from colonies with the lowest calculated breeding values in our sample (Additional file 8: Table S8). The offspring of those three crosses established the workers in the low HB source group in our experiment. Five drones derived from different mothers and grandfather (not related) one drone had the some mother and grandfather (a replicate). Crosses were performed by instrumental insemination using the semen from the different, single drones.

### Behavioral assay

Hygienic behavior (HB) was evaluated in individualized age-standardized worker bees (the progeny of the F1 generation, see Fig. 1) using pin-killed brood (the so called “pin-test”). Directly after eclosion, the worker bees were individually labeled with small colored, numbered tags (Opalith-Plättchen) assembled in equal proportions (~300 individuals per backcross line, ~1800 altogether) and kept in a queen right host colony until the bees reached the age of 12 days, which is the period of time during which worker bees engage in HB at the highest frequency [34]. Throughout the experiment, the worker bees were kept as a separate group using a gauze mesh cage that was supplied with honey and pollen, which was inserted into the queen right colony. The worker bees had physical and olfactory contact with the host colony which was standard colony which was not involved in the selection program. At the onset of the experiment, the worker bees were transferred to an experimental comb supplied with brood, honey and pollen, in which an average of 21.5 pupae in sealed brood cells within a square of 11 × 10 cells had been pierced at regular intervals; approximately 90 non-pierced pupae in sealed brood cells were used as non-treated controls. Brood combs for the pin-test assay were derived from several randomly chosen colonies. The experimental comb was inserted into a cage with a glass front on one side, opposite of the manipulated area, and a gauze mesh on the other side, allowing for physical and olfactory contact with the host colony. The glass front of the cage faced an infrared-sensitive camera [22]. During the experiment, the comb was illuminated with infrared light, a wavelength that the bees cannot perceive, allowing them to behave as they would in the natural darkness of the hive (LEDs: OSA Opto-Light GmbH, Germany, Type: OIS 330,880). The experiment lasted 12 h, during which the movements of the worker bees on the brood area were monitored by video recording using an infrared-sensitive camera (Panasonic WV-NP1004 megapixel color network IP). Directly after the experiment, the worker bees were anaesthetized with CO_2_, shock-frozen in liquid nitrogen and stored at −70 °C until further analysis. The experiment was replicated four times with another set of worker bees acquired from the six crosses. Manual analysis of the video recordings enabled the scoring of the HB (comprising uncapping of sealed brood cells and removal of dead brood) of individual workers. Any HB that a single worker bee performed once or several times at one or more cells was scored. Bees that performed HB at the sealed control cells were omitted from further analysis. Worker bees that displayed no HB were detected as follows: worker bees that were present in the observed brood area were registered in time frames of 5 min. Worker bees that were repeatedly observed in the brood area and possibly could percept the stimulus of the dead pupae but did not display HB were scored as non-hygienic. The numbers of uncapped cells and removed brood from the pin-treated brood cells greatly exceeded the number of uncapped cells and removed brood for the non-treated cells (*χ* ^*2*^-test, *P* < 0.0001) in our behavioral analyses. Although we cannot exclude the possibility that some control cells contained diseased brood, this result demonstrated that HB was specifically induced by pin treatments in our behavioral assays. We applied the non-parametric Mann–Whitney U-test (MWU-test) and the *t* test to compare differences in HB. Statistical analyses were performed using PASW Statistics 18 (SPSS Inc., Illinois) software.

### Microarray analysis

The honeybee whole-genome oligonucleotide microarray (Design: UIUC Honey bee oligo 13 K v1, Accession: A-MEXP-755) contains 28,800 oligos representing 13,440 genes derived from annotations of the entire honeybee genomic sequence [18]. A total of 82 microarrays were used for the profiling of the samples (Additional file 1: Table S1). We randomly chose bees from each backcross and experimental replicate. Total RNA was isolated from individual brains (including the optical lobes, excluding the retina) using a standard TRIzol protocol with subsequent purification by filter columns (Qiagen, Germany) and column removal of DNA by DNase digestion. A total of 1 µg of RNA was amplified prior to labeling following the manufacturer’s protocol (MessageAmp II aRNA Amplification kit, Ambion). We hybridized 3 µg of each labeled RNA sample to a single microarray slide. The slides were scanned (Axon 4000B Scanner), and raw hybridization signals were extracted (GenePix Pro 6.0 software, Agilent Technologies). Transcription-level data were processed and analyzed using the LIMMA 2.16 software package (https://www.bioconductor.org/packages/3.3/bioc/html/limma.html). The quality of hybridization was evaluated using the raw expression data from control probes spotted on each slide. Transcription-level data were corrected for background signal (“normexp” function) [35] and intensity-dependent bias was detected (“normalize within arrays” function with the default print-tip lowess normalization method) [36]. Finally, the log-transformed expression ratios were calculated. Data from duplicate spots were averaged using the “avedups” function. We used a design matrix that incorporated the behavior and HB source conditions and linear models using the Bayesian fitting option. All microarray data are MIAME-compliant, and the raw data have been deposited in a MIAME-compliant database (ArrayExpress, EMBL-EBI). Differences in gene transcription that resulted from behavior or from the type of backcross were specified as separate contrasts using linear models. *P* values were adjusted for multiple testing with a 5 % false discovery rate. Functional annotation of gene sets that fell into similar categories of GO terms for molecular processes and biological functions were identified using DAVID (http://david.abcc.ncifcrf.gov/) [24, 25], which includes an enrichment analysis of GO terms. We used the gene annotations from the UIUC Honey Bee oligo 13 Kv1 annotation file.

## Additional files

**Additional file 1: Table S1.** The design of the microarray experiments that comprise 82 hybridizations.

**Additional file 2: Table S2.** DEGs associated with hygienic behavior (HB) in the high HB source. A modified t-test was performed on the log-transformed transcription ratios gained from 82 hybridizations between 138 hygienic worker bees and 138 non-hygienic siblings. Oligo Ids, Gene Ids, and Comments are quoted from the UIUC Honey bee oligo 13 K v1 annotation file (May 2007).

**Additional file 3: Table S3.** DEGs associated with hygienic behavior (HB) in the low HB source. A modified t-test was performed on the log-transformed transcription ratios gained from 82 hybridizations between 138 hygienic worker bees and 138 non-hygienic siblings. Oligo Ids, Gene Ids, and Comments are quoted from the UIUC Honey bee oligo 13 K v1 annotation file (May 2007).

**Additional file 4: Table S4.** DEGs associated with the HB sources but not with the behavior. A modified t-test was performed on the log-transformed transcription ratios gained from 82 hybridizations. Oligo Ids, Gene Ids, and Comments are quoted from the UIUC Honey bee oligo 13 K v1 annotation file (May 2007).

**Additional file 5: Table S5.** The enrichment analysis for HB associated DEGs from the high HB source. Enrichments above the score 2.0 are presented.

**Additional file 6: Table S6.** The GO enrichment analysis for HB associated DEGs from the low HB source. Enrichments above score 2.0 are presented.

**Additional file 7: Table S7.** List of HB associated DEGs from high and low sources found in previous studies.

**Additional file 8: Table S8.** The breeding values of HB for the drones.

## Abbreviations

HB: hygienic behavior
DEG: differently expressed genes
VSH: Varroa sensitive hygiene
QTL: quantitative trait loci
MWU test: Mann–Whitney-U test
GO: gene ontology
BLUP: best linear unbiased method
