# Supplementary material for: Behavioral and molecular studies of quantitative differences in hygienic behavior in honeybees
Source: BMC Res Notes. 2016 Oct 21;9:474. doi: 10.1186/s13104-016-2269-y (PMC5073793; doi:10.1186/s13104-016-2269-y)
Supplement: Supplementary file 7 — Additional file 7: Table S7. List of HB associated DEGs from high and low sources found in previous studies. [file 13104_2016_2269_MOESM7_ESM.docx]

**Table S7:**

List of HB associated DEGs from high sources that were found in previous studies

| **This transcriptome study** | | | **Listed in study** |
| --- | --- | --- | --- |
| **Honey bee gene ID** | **Gene description** | **Putative function** |  |
| GB12219 | similar to G protein pathway suppressor 1 (LOC409357) | COP9 Signalosome  Cell cycle | Tsurada 2012 |
| GB19123 | prostaglandin E2 receptor EP4 subtype-like | regulation of Rhoprotein signal transduction | Tsurada 2012 |
| GB18005 | similar to Es2 CG1474-PA (LOC412314) | RNA splicing | Oxley  2010 |
| GB15141 | Dscam exon 10.9 | Neuronal function | Gempe 2012, Navajas 2008, LeConte 2011 |
| GB19503 | similar to hsp 8 (LOC410620) | Stress response, Chaperon | Navajas 2008, Mondet 2015 |
| GB11499 | similar to CG31004-PA (LOC725498) | cell-matrix adhesion | LeConte 2011 |
| GB30209 | Dscam exon 15 | Neuronal function (mushroombody development) | LeConte 2011 |
| GB20104 | similar to germinal histone H4 (LOC725230) | chromatin | Mondet 2015 |
| GB13705 | Prm1 | Spermiogenese, cell fusion, chromatin condensation | Mondet 2015 |
| GB14159 | similar to CG31142-PA (LOC726160) | - | Mondet 2015 |
| GB18010 | nitric oxide synthase (NOS) | divers | Mondet 2015 |
| GB14494 | similar to hsp1, alpha (LOC411700) | Stress response | Mondet 2015 |
| GB11097 | Tret1-like | carbohydrate transport | Mondet 2015 |
| GB13787 | similar to Task6 CG9637-PA (LOC411036) | Transmembrane transport | Mondet 2015 |
| GB20070 | alpha-glucosidase (Hbg2) | carbohydrate metabolic process | Mondet 2015 |
| DB729986 | cDNA library, head Apis mellifera clone BH10005O14 | - | Gempe 2012 |
| GB14163 | similar to CG11533-PA, isoform A (LOC411575) | - | Gempe 2012 |
| GB11297 | Serinprotease 31 | regulation of melanization defense response | Gempe 2012 |
| DB754387 | cDNA library, head Apis mellifera clone BH10093C06 | - | Gempe 2012 |
| GB18555 | similar to CG33722-PC, isoform C (LOC727144),  tether containing UBX domain for GLUT4 | ? | Gempe 2012 |
| GB30197 | similar to CG18497-PA, isoform A (LOC412243) | - | Gempe 2012 |
| AM12884 | uncharacterized | - | Gempe 2012 |
| GB19093 | similar to Guanine nucleotide-binding protein G(o) subunit alpha 47A (LOC726858) | withdrawn | Gempe 2012 |
| GB10720 | similar to casein kinase 1, alpha 1 (LOC410129) | DNA repair, cell division, nuclear localization ,membrane transport | Gempe 2012 |
| GB18639 | Mediator complex subunit 10 CG5057-PA (LOC552403) | regulation of transcription from RNA polymerase II promoter | Gempe 2012 |
| GB18773 | similar to dally-like | Neuronal function, segment polarity determination | Gempe 2012 |
| GB16236 | uncharacterized | - | Gempe 2012 |
| GB14642 | IGFn3-1 | - | Gempe 2012 |
| GB18519 | uncharacterized | - | Gempe 2012 |
| GB30201 | uncharacterized | - | Gempe 2012 |
| BB170001B10C03.5 | Bee Brain Library, BB17 Apis mellifera cDNA clone | - | Gempe 2012 |
| GB13106 | uncharacterized | - | Gempe 2012 |
| NW_001257055.1 | genomic contig, reference assembly | - | Gempe 2012 |
| DB761450 | cDNA library, head Apis mellifera clone BH10023N15 | - | Gempe 2012 |

List of HB associated DEGs from low sources that were found in previous studies

| **This transcriptomic study** | | | **Listed in study** |
| --- | --- | --- | --- |
| **Honey bee gene ID** | **Gene description** | **Putative function** |  |
| GB11986 | similar to Protein still life, isoform SIF type 1 (LOC725970) | signal transduction, regulation of synapse structure and  activity | Tsurada 2012 |
| GB11159 | uncharacterized | - | Tsurada 2012 |
| GB11135 | odorant binding protein 1 | Odorant binding | Oxley  2010 |
| GB19838 | uncharacterized | - | Oxley  2010 |
| GB12929 | sodium channel protein paralytic | Response to mechanical stimulus | Navajas 2008 |
| GB15141 | Down syndrome cell adhesion molecule | Neuronal function (mushroombody development) | Navajas 2008, LeConte 2011 |
| GB10036 | defensin 2 (LOC413397) | Antimicrobial peptide | Mondet 2015 |
| GB19733 | f-box/LRR-repeat protein 21-like | Protein ubiquitination | Mondet 2015 |
| GB14620 | histone H3-like | chromatin | Mondet 2015 |
| GB14742 | agrin-like | Cell morphogenese | Gempe 2012, Mondet 2015 |
| GB19709 | similar to Syt7 CG2381-PG (LOC409138) | Neurotransmitter secretion | Gempe 2012 |
| BB170030B10C12.5 | Bee Brain Library, BB17 Apis mellifera cDNA clone | - | Gempe 2012 |
| GB18344 | hypothetical protein LOC726543 (LOC726543) | - | Gempe 2012 |
| NW_001253489.1 | linkage group 7 genomic contig, reference assembly | - | Gempe 2012 |
| GB18362 | protein inhibitor of activated STAT | Gene expression, DNA repair | Gempe 2012 |
| BB170014B20E03.5 | Bee Brain Library, BB17 Apis mellifera cDNA clone | - | Gempe 2012 |
| GB55755 | outspread | - | Gempe 2012 |
| BB170030B20H03.5 | Bee Brain Library, BB17 Apis mellifera cDNA clone | - | Gempe 2012 |
| BB170020B20F11.5 | Bee Brain Library, BB17 Apis mellifera cDNA clone | - | Gempe 2012 |
| BB170027A10E07.5 | Bee Brain Library, BB17 Apis mellifera cDNA clone | - | Gempe 2012 |
| GB18664 | ubiquitin-conjugating enzyme E2 R2-like | Protein mediated proteolyses | Gempe 2012 |
| BB170019B20H10.5 | Bee Brain Library, BB17 Apis mellifera cDNA clone | - | Gempe 2012 |
| GB17926 | CUGBP Elav-like family member 4-like (CELF4) | RNA-splicing, editing, translation | Gempe 2012 |
| DB745343 | cDNA library, head Apis mellifera clone BH10061K19 | - | Gempe 2012 |
| BB170021A20F02.5 | Bee Brain Library, BB17 Apis mellifera cDNA clone | - | Gempe 2012 |
| BB170012A20D05.5 | Bee Brain Library, BB17 Apis mellifera cDNA clone | - | Gempe 2012 |
| DB747211 | cDNA library, head Apis mellifera clone BH10068A21 | - | Gempe 2012 |
| BB160013B20B06.5 | Bee Brain Library, BB16 Apis mellifera cDNA clone | - | Gempe 2012 |
| NW_001253238.1 | linkage group 1 genomic contig, reference assembly | - | Gempe 2012 |
