# Supplementary material for: Behavioral and molecular studies of quantitative differences in hygienic behavior in honeybees
Source: BMC Res Notes. 2016 Oct 21;9:474. doi: 10.1186/s13104-016-2269-y (PMC5073793; doi:10.1186/s13104-016-2269-y)
Supplement: Supplementary file 8 — Additional file 8: Table S8. The breeding values of HB for the drones. [file 13104_2016_2269_MOESM8_ESM.docx]

Table S8. The breeding values of HB for the drones.

| Queen | Drone | | HB source |
| --- | --- | --- | --- |
| # | # | HB breeding value ^1)^ |  |
| 4/1/23 | 18/1/74 | 119 | High |
| 4/1/27 | 18/1/74 | 119 | High |
| 4/1/31 | 18/1/74 | 119 | High |
| 4/1/55 | 18/6/5 | 104 | Low |
| 4/1/59 | 8/1/463 | 86 | Low |
| 4/1/64 | 8/1/463 | 86 | Low |

1) HB breeding value gives the relative breeding value for HB colony performance in the population. A value of 100 would denote the population average of the breeding population.
